# Axillary artery access for cardiac interventions in newborns

**DOI:** 10.4103/0974-2069.43878

**Published:** 2008

**Authors:** Dietmar Schranz, Ina Michel-Behnke

**Affiliations:** Pediatric Heart Center Justus Liebig University, Giessen, Germany; 1 Department of Pediatric Cardiology, Medical University of Vienna, Austria

**Keywords:** Axillary artery, duct stenting, valvuloplasty, coarct dilatation

## INTRODUCTION

Vascular access is an extremely important issue for successful interventional therapy in critical congenital heart diseases, particularly in the newborns. The axillary artery access for demanding interventions in newborns is rarely described, despite its many obvious advantages.[1–3] Two major types of congenital heart diseases that might be treated with an improved success rate by axillary access are: newborns with duct-dependent pulmonary blood flow having a critically or totally obstructed ventricular-pulmonary connection particularly with a tortuous duct inserting at the inner curve of the aortic arch opposite to the origin of the left or the right subclavian artery; and the neonates or even premature newborns with critical aortic valve stenosis with or without aortic coarctation.

In neonates with critical aortic stenosis combined with aortic coarctation, the right axillary artery approach is a favorable alternative to a femoral or even a carotid artery access. The use of the femoral arterial access has a high risk of significant vessel damage particularly in a premature or newborns suffering from aortic coarctation with impaired flow to the lower part of the body. We favor the axillary artery access to the carotid artery approach which needs a surgical cut down and repair.[4]

In patients with duct-dependent pulmonary blood flow, duct stenting is increasingly used as a minimally invasive alternative to a modified Blalock-Taussig shunt (mBTS), which is still the most commonly used systemic-pulmonary artery shunt in neonates with cyanotic heart disease. Surgery in neonates, particularly in premature infants, is associated with major complications despite improvement in the surgical techniques as well as the intensive care.[5,6] Morbidity and mortality after mBTS are related to several factors including age, pulmonary artery diameter, and the baseline cardiac anatomy.[5,6] In 1991, the first experimental use of an intravascular stent to maintain ductal patency in an animal model was reported.[7,8]

In the context of the current knowledge and literature[3] and also based on our own experience of ductal stenting in almost 70 patients with duct-dependent pulmonary circulation, this paper deals with the technical aspects of axillary artery access including risk stratification and possible limitations and complications of this approach.

## CARDIAC CATHETERIZATION

Prior to duct stenting or balloon valvuloplasty in a newborn with critical aortic valve stenosis or severe coarctation as first-stage palliation, a detailed diagnosis needs to be established by two-dimensional echocardiography and Doppler. In those with complex cardiac anatomy, it is essential to categorize them as having either single-ventricle or two-ventricle physiology. With duct-dependent circulation, it is essential to define the exact duct morphology and its relationship to the aortic arch. Depending on the institutional experience, procedures are performed under general anesthesia or conscious sedation. In hemodynamically stable patients, we prefer spontaneous breathing. Mostly, diazepam (0.2–0.5 mg/kg) and ketamine (0.2–0.5 mg/kg) in small, intermittent doses are sufficient. In case of prostaglandin therapy, continuous infusion in lowest possible maintenance dose (PGE1; 5–20 ng/kg/min) is recommended to avoid apnea. Prophylactic antibiotic treatment (cefuroxime in most patients) is usually advisable. Attention to bleeding during catheter and wire manipulation is extremely important. After creation of vascular access and placement of the sheaths, intravenous heparin is routinely administrated, 100 U/kg as a single dose for duct stenting and 50 U/kg in case of valvulo- or angioplasty. Following successful stent placement, heparin is given continuously in a dose of 300 U/kg over a minimum of 24 hours. Aspirin as cyclo-oxygenase inhibitor is not given routinely and clopidogrel at 0.2 mg/kg/day is used only in some selected cases.

## AXILLARY ARTERY ACCESS

**Figure 1:**
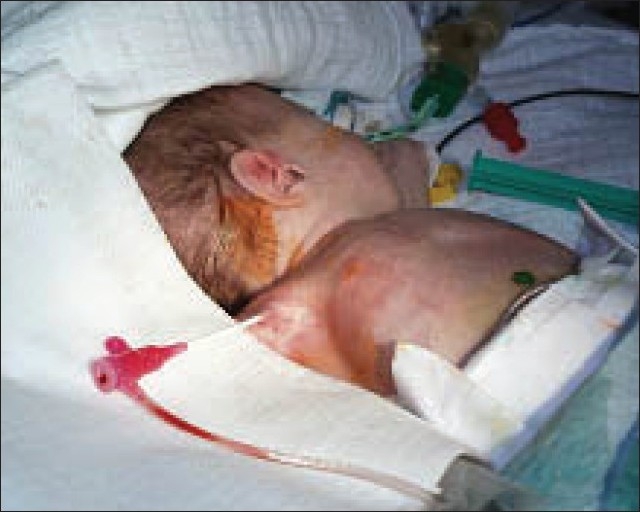
Premature baby with critical aortic valve stenosis combined with critical aortic coarctation. The baby was admitted in cardiogenic shock; intubation and controlled ventilation was necessary before a lifesaving interventional therapy was performed by using a right-sided axillary access

In newborns with critical aortic valve stenosis or critical coarctation, or when there is a combination of both, right axillary artery access is often the access of choice [Figure 1]. Vascular access for duct stenting in newborns with duct-dependent pulmonary circulation depends on the insertion of the duct in relation to the aortic arch, the anatomical considerations of the congenital heart defect, and the availability of the hardware. For stenting a duct originating from the inner curve of the transverse aortic arch, axillary access on the ipsilateral side just opposite to the duct insertion is very useful. In most cases, the anatomical relationship of duct insertion to head-neck arteries can be determined by echocardiography, only in a few patients the exact positioning of the duct insertion needs to be determined by an additional angiography.

**Figure 2:**
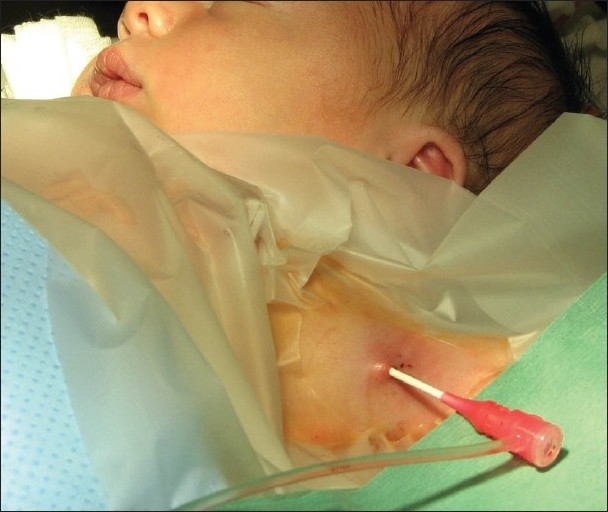
4F Terumo sheath placed in the left axillary artery of a premature baby with pulmonary atresia with ventricular septal defect in whom duct stenting was performed successfully

Figure 2 shows the left axillary access for duct stenting in a premature baby with pulmonary atresia with ventricular septal defect. In most patients, a 4F sheath can be placed after direct puncture of the axillary artery; surgical cut down is only rarely necessary.

**Figure 3:**
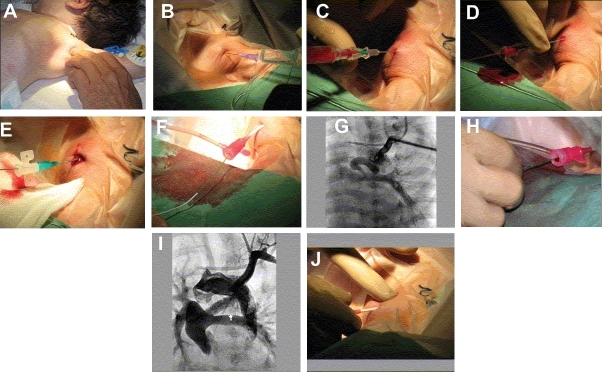
(A) Gentle palpation of the left axillary artery in a deeply sleeping, nonintubated newborn with complete transposition of the great arteries with severe left ventricular outflow tract obstruction prior to duct stenting. The head is slightly turned to the right, the angle of left upper limb to thorax has to be about 120–140°; an extreme extension of the left arm leads to tension on the axillary area, which might be unfavorable for direct puncture of the artery. (B) Subcutaneous infiltration of local anesthesia exactly in the area of planned vessel puncture. Avoid too much amount of anesthetic fluid, which might make the axillary artery impalpable; repetitive infiltration after sheath placement is preferred. (C) The axillary artery is punctured by using a 21G needle (Vygon, Aachen Germany). We prefer to puncture the vessel by applying a continuous low negative pressure with the help of a 2 ml syringe connected to the needle. (D) After successful arterial puncture, a 0.018-inch soft wire is advanced through the needle within the vessel (Seldinger technique); in some situations, a 0.014-inch floppy wire can be advanced more easily. It is important to avoid excessive bleeding at this stage. (E) A standard arterial catheter (Leader Cath, Vygon), which is smoothly advanced over the wire at first, and placed within the subclavian artery. After confirming its position by contrast injection, a short 0.021-inch guidewire is placed through the arterial catheter under fluoroscopy over which a 4F sheath (Terumo) is subsequently introduced within the artery. (F) A 4F Cobra catheter is advanced through the 4F terumo sheath over a 0.035-inch guidewire. (G) An angiography performed through the 4F sheath placed in the subclavian artery depicted an extremely tortuous ductus arteriosus which seems to be obstructed at the pulmonary end. (H) A premounted coronary stent (Driver, Medtronic), was advanced through the 4F sheath over a 0.014-inch floppy wire which was already positioned through the PDA into the pulmonary artery. (I) A final angiogram after successful duct stenting; the angiography was also performed through the 4F sheath by hand-injection of contrast medium. (J) Following duct stenting, and while removing the 4F sheath, careful compression of the axillary artery is mandatory to avoid post-interventional bleeding. The compression has to be as long as the arterial bleeding is completely stopped, a continuous compression is not further necessary and the patient can be transferred back to the ward/ICU. The site of puncture is to be observed closely during transport and after the child is shifted to the ward/ICU

In Figures 3A–J, detailed procedural steps utilizing left axillary access for duct stenting are described in a newborn with transposition of the great arteries and associated subpulmonary, left ventricular outflow tract obstruction.

## MATERIALS

### Axillary access for duct-dependent pulmonary circulation

1. Arterial puncture set (20G/2.7F; Leader Cath, Vygon)
2. 4F short sheath, 7 cm (Terumo), axillary artery access
3. Cobra 4F catheter (Terumo, Cordis)
4. Berman angiographic catheter 4F
  Pigtail catheter 4F 0.035-inch lumen
  Judkins R 4F, 0.035-inch lumen
5. Balloon-catheter 3 × 20 mm (Apex™Boston Scientific)
6. 0.014 floppy guidewire (BMW soft, stiff support Abbott)
7. 0.035 guidewire
8. Stents: Driver™(Medtronic), Liberé ™ (Boston Scientific), 4 (3) mm widths, lengths 9, 12, 15, 18, 22, 24 mm

### Axillary access for aortic valvuloplasty or aortic angioplasty

1. Arterial puncture set (Leader Cath, Vygon)
2. 4F short sheath, 7 cm (Terumo), axillary artery access
3. Cobra 4F catheter (Terumo, Cordis)
4. Berman angiographic catheter 4F
  Pigtail catheters 4F 0.035-inch lumen
  Judkins R 4F, 0.035-inch lumen
5. Balloon-catheter 3 or 4 × 20 mm (Apex™Boston Scientific)
  Balloon-catheter 5–8 × 20/30 mm (Sterling™Boston Scientific)
6. 0.014 floppy guidewire (BMW soft, stiff support Abbott)
  0.035 guidewire

### Checklist to avoid complications and pitfalls

1. Well-defined long-term therapeutic goal (biventricular or univentricular repair), and precise planning of the strategy for the intervention.
2. Written informed consent of the parents based on a consensus with the pediatric cardiac surgeon; in selected cases surgical stand by.
3. Adequate axillary-vascular access in context of the pathophysiology, patient's size, ductal origin from the aortic arch (vertical!).
4. In duct-dependent patients, precise evaluation of duct morphology (tortuous, obstructed, or unobstructed), exact determination of the duct length, assessment of the narrowest as well as widest part, and evaluation of the pulmonary as well as aortic end of the duct is mandatory.
5. After deciding to use either the right or left axillary access, the axillary area has to be carefully prepared. The axilla, is well exposed by fixing the arm in head-up position of about 120–140°. The puncture area is exactly defined by finger palpation or by Doppler in a well-sedated patient. Careful injection of local anesthesia in the lowest effective dose is very important to avoid losing the landmark of axillary artery. The axillary artery is punctured by applying a minimal negative pressure on the 2 ml syringe fixed on the needle. Once a free flow of arterial blood is obtained, we gently advance a standard 0.018-inch guidewire or alternatively a 0.014-inch floppy guidewire is helpful to avoid intimal dissection. After confirming the wire placement under fluoroscopy, an atraumatic 4F sheath is gently slid over the guidewire.
6. Before proceeding further with catheterization, it is important to check free blood flow or even make an injection of a small amount of contrast medium through the sheath to exclude an intimal lesion or extra-luminal sheath position in the false lumen. A small shot of contrast medium through the 4F sheath delineates mostly the anatomy of the target lesion. Left lateral projection is used in newborns with critical aortic valve stenosis or even coarctation and a combined left anterior oblique 30° and cranial 20° mono-plane projection in duct-dependent newborns with pulmonary valve obstruction.
7. A floppy guidewire is placed across the target vessel or the aortic valve through a 4F Cobra or 4F right Judkins catheter.
8. In order to avoid bleeding complication after the intervention, the sheath is removed gently and the punctured site is pressed with optimum force. Due to the limited subcutaneous fat as well as the short distance from the site of the punctured artery to the skin, a continuous observation of the access area is mandatory, even later on when the baby is back to the ward.

## DISCUSSION

Axillary access for interventions in newborns with complex or critical congenital heart diseases is a real alternative to a more commonly used femoral or even carotid artery access. There are many obvious advantages of such an access which have been rarely described.[1–3] The most important advantage of this access is that the axillary artery is not an end-artery and therefore does not have the disadvantage of cannulating such an artery. While using the axillary artery, the arm continues to be perfused by the second intercostal artery as well as by the acromial artery. Moreover, the axillary artery is better felt in the smaller patients, including the premature newborns.[9] especially in those suffering from critical coarctation with low cardiac output and nonpalpable femoral pulses. In this subset of patients, there is a high risk of vascular complications after puncture and cannulation, due to a low-flow situation.

Optimum analgesia and sedation, which avoids apnea in prostaglandin treated newborns, combined with smooth local anesthesia allows such demanding interventions in spontaneously breathing babies. This makes interventional approach less invasive as compared to standard surgical procedures.

The success rate for using the axillary access is greatly influenced by a careful preparation of the axillary area. The procedure has to be performed in a well-sedated patient in whom the upper limb has to be placed in a head-up position without inducing too much tension on the axillary area. The head is turned slightly to the opposite side in such a way that the axillary artery still remains palpable. A gentle infiltration of local anesthesia in a small amount is necessary to avoid losing the landmark for the site of puncture. If the axillary artery is not well palpable, there are no other anatomical landmarks available for a “blind puncture”. Therefore, when the axillary artery is not palpable puncturing by Dopple guidance may be the only realistic alternative for a successful intervention. The left axillary artery access is more difficult than the right because of the operator being unfamiliar to standing on the left of the patient. Also, the position to the X-ray tube as well as the monitors is a little cumbersome and manipulating catheters and wires is difficult for a right-handed individual. These limitations can be overcome with experience. Till such time, it is advisable to have four rather than two experienced hands.

For (premature) newborns with critical aortic stenosis with or without aortic coarctation, right axillary access has to be considered as an alternative to a femoral or even carotid artery access.

In patients with duct-dependent pulmonary flow, the site of access depends mainly on the location of the ductus. Therefore, detailed diagnosis has to be established by two-dimensional and Doppler echocardiography for planning the interventional strategy. A “normal” duct arising from the descending aorta or the one arising from the subclavian artery can be relatively easily engaged by using a 4F Judkins right coronary or Cobra-shaped catheter. Retrograde duct stenting via the femoral artery is then the preferred access using a 4F long-sheath (Cook) or 25 cm long 4F sheath (Terumo). When the duct originates from the inner curve of the transverse aortic arch which is more often the case, femoral access is still feasible utilizing a 4F pigtail catheter with its loop cut to give an “inverted J” which can be used to engage the ductal ampulla. Alternatively, it may be possible to access the aorta antegradely from the femoral vein through the heart utilizing a right Judkins-shaped 5F guiding catheter or 4F long sheath, but this route makes catheter control more difficult and may cause hemodynamic instability in small neonates by keeping the atrioventricular and semilunar valves open.

However in cases with a vertical duct, we prefer an access via the axillary artery route, as previously described in detail. Depending on the position of the duct at the inner curve of the aortic arch in relation to the subclavian arteries, which can mostly defined prior to the intervention by echocardiography, the ipsilateral left or right axillary artery is used. Direct puncture of the axillary artery is preferred for placing a 4F sheath (Terumo) within the subclavian artery. It is important to confirm the position of the sheath within the true arterial lumen by injecting a small amount of contrast before proceeding with further catheterization. A floppy-tipped 0.014-inch coronary guidewire is placed through the duct in the distal pulmonary artery branch or looped in the main pulmonary artery using a 4F Judkins or Cobra catheter for wire guiding. Although, positioning the wire via axillary approach is simple, it needs to be done carefully to avoid trauma to any of the vascular structures. With increasing experience and gentle manipulations, many severe complications of duct stenting can be avoided. Stenting the long tortuous ducts is not only facilitated by the use of low-profile coronary stents with high longitudinal flexibility, high conformability, minimal foreshortening, and minimal recoil but also by the use of the axillary access which is more straight as compared to the femoral access which involves an additional curve during duct stenting. Manipulations of the arm for advancing a premounted stent was not necessary in our experience, but replacement of a wire or exchange for a stiffer wire or even predilation of a stenosed duct were necessary in some cases prior to placement of a stent. A small quantity of contrast is hand injected through the sheath for optimum stent positioning.

Considering potential complications of the axillary approach such as an arterial dissection (*n* = 1), formation of a hematoma (*n* = 2), excessive bleeding (*n* = 2), and nerve palsy (*n* = 0), we are still convinced that with increasing experience and by meticulously following the step-by-step approach that we have described, complications by the axillary access can be minimized or even avoided.

In summary, the axillary access is an attractive, alternative approach to treat newborns with complex heart diseases. In an elective procedure, if one is not successful in getting an axillary access, it is advisable to stop the intervention. Considering the rule “*primum nil nocere*”, a second attempt one or several days later is not a shame.
